# Supplementary material for: Saccharomyces boulardii (CNCM I-745) improves intestinal damage in sepsis by remodeling intestinal flora structure
Source: Front Cell Infect Microbiol. 2025 Jul 31;15:1602792. doi: 10.3389/fcimb.2025.1602792 (PMC12350329; doi:10.3389/fcimb.2025.1602792)
Supplement: Supplementary file 1 [file DataSheet1.zip › summary/summary/7_Differential_analysis/1_diff_abundance/7_Species/Group.DW0_vs_CW0_vs_BW0_vs_AW0/Group.DW0_vs_CW0_vs_BW0_vs_AW0_diff_barplot.pdf]

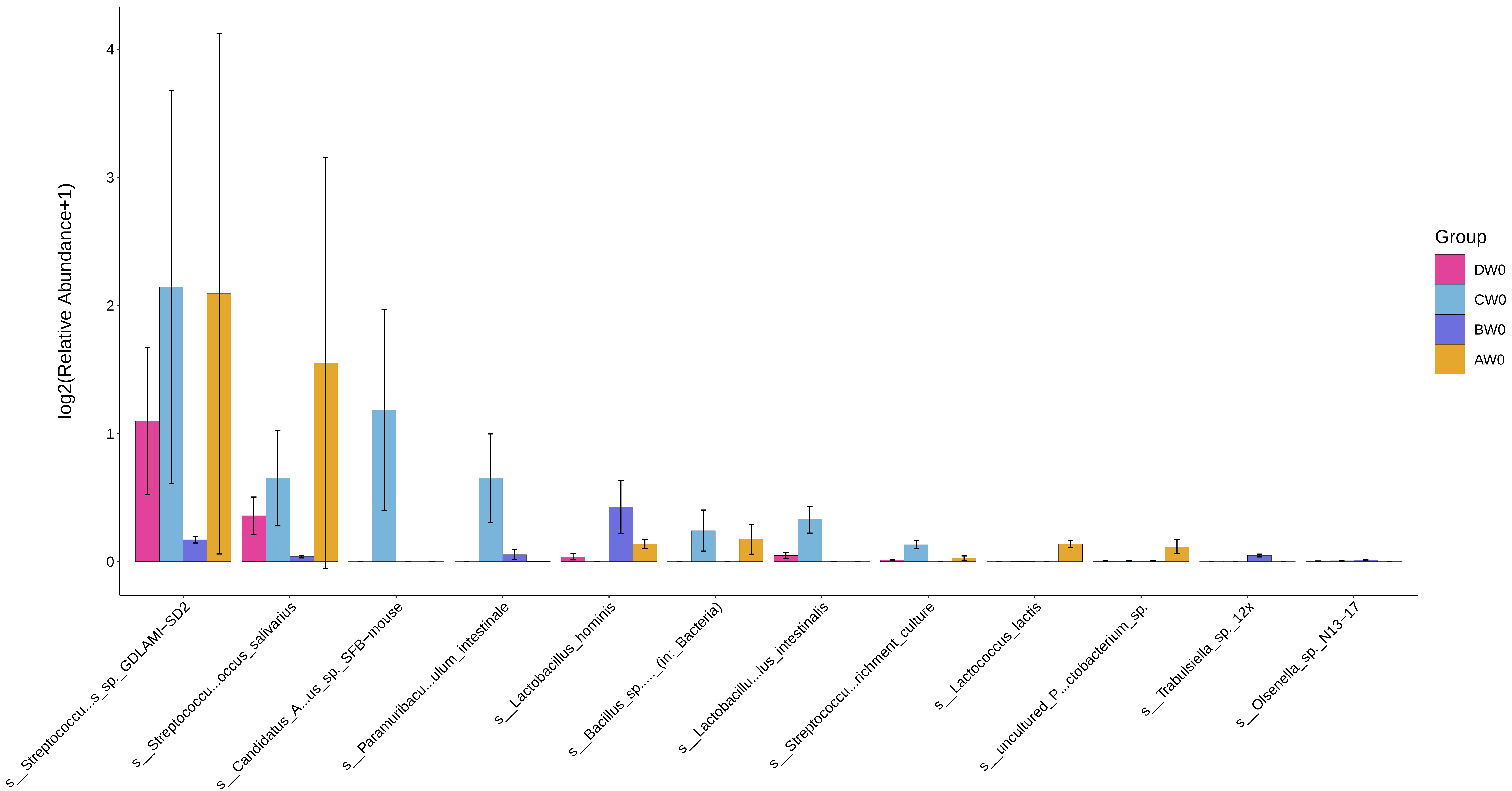

log2(Relative Abundance+1)
0
1
2
3
4
Group
DW0
CW0
BW0
AW0
s__Streptococcu...s_sp._GDLAMI−SD2
s__Streptococcu...occus_salivarius
s__Candidatus_A...us_sp._SFB−mouse
s__Paramuribacu...ulum_intestinale
s__Lactobacillus_hominis
s__Bacillus_sp.…_(in:_Bacteria)
s__Lactobacillu...lus_intestinalis
s__Streptococcu...richment_culture
s__Lactococcus_lactis
s__uncultured_P...ctobacterium_sp.
s__Trabulsiella_sp._12x
s__Olsenella_sp._N13−17
